# Identification of Polish cochineal (*Porphyrophora polonica* L.) in historical textiles by high-performance liquid chromatography coupled with spectrophotometric and tandem mass spectrometric detection

**DOI:** 10.1007/s00216-016-9408-0

**Published:** 2016-03-02

**Authors:** Katarzyna Lech, Maciej Jarosz

**Affiliations:** Faculty of Chemistry, Chair of Analytical Chemistry, Warsaw University of Technology, Noakowskiego 3, 00-664 Warsaw, Poland

**Keywords:** Polish cochineal, Natural dyes, Anthraquinones, HPLC, Tandem mass spectrometry, Historical textile analysis

## Abstract

The present work reports a method for identification of Polish cochineal (*Porphyrophora polonica* L.) in historical fabrics by the use of high-performance liquid chromatography coupled with diode array and tandem mass spectrometric detection with electrospray ionization (HPLC-DAD-ESI MS/MS). This hyphened technique allows detection and identification of 16 new minor colorants present in the discussed scale insect (including two previously observed by Wouters and Verhecken (Ann Soc Entomol Fr. 1989;25:393–410), but specified only as compounds of unknown structures) that do not occur (e.g., in American cochineal). The MS/MS experiments, complemented with UV–VIS data, enable identification of mono- and di-, *C*- and *O*-hexosides of kermesic and flavokermesic acids or their derivatives. The present paper introduces a fingerprint of color compounds present in Polish cochineal and defines them, particularly pp6 (ppI, *O*-hexoside of flavokermesic acid), as its markers allow distinguishing of Polish-cochineal reds from the American ones. Usefulness of the selected set of markers for identification of Polish cochineal has been demonstrated in the examination of textiles from the collection of the National Museum in Warsaw using the multiple reaction monitoring (MRM) method, originally elaborated on the basis of this study.

**Electronic supplementary material:**

The online version of this article (doi:10.1007/s00216-016-9408-0) contains supplementary material, which is available to authorized users.

## Introduction

Polish cochineal (*Porphyrophora polonica* L.) is a scale insect that used to be fairly widespread over the Palearctic region (mainly Poland, Ukraine, Belarus, Germany, and Czech Republic, but also France, Hungary, Switzerland, Russia, Kazakhstan, Kyrgyzstan, and even Mongolia and China) [1, 2]. For centuries, Polish cochineal was the most important source of the European red dye with a very long and interesting history of use, but currently it is considered an extremely rare species, and hence it is protected in many countries [1]. It is most probably the reason why this dye, obtained from Polish cochineal, has not been studied for the last 25 years.

The red dye obtained from Polish cochineal was known in Europe and the Mediterranean countries since antiquity [3], but it gained importance in the Middle Ages [2]. In the 15th and in the first half of the 16th century it was exported from Poland to Italy, the Netherlands, France, England, Turkey, and even to Armenia [1], but in the end of the 16th century it was replaced in many of the leading weaving centers by *Dactylopius* species imported to Europe from Central and Southern America [3]. In the following centuries, Polish cochineal was used only in local textile workshops of Central Europe with direct access to the raw material [4]. Therefore, information on its occurrence in fabrics could be very helpful in determining their origin or dating.

As a natural dye, the red from Polish cochineal is a mixture of colorants, the composition of which is very similar to other animal red dyes such as American or Armenian cochineals. They all contain mainly carminic acid and many minor colorants (i.e., flavokermesic acid, kermesic acid, dcII, dcIV, dcVII) [2, 3]. Therefore, their proper identification requires preliminary separation of compounds with the use of high-performance liquid chromatography (HPLC).

The first studies of Polish cochineal by HPLC were carried out with spectrophotometric detection at 275 nm in the 1980s [5, 6]. These works showed that colorant compositions of *P. polonica* L. and *P. hamelii* Brandt (two closely related species) are very similar. Moreover, due to the limited availability of Polish cochineal caused by decrease in its population and its protection by law, the content of the main colorants determined by the authors of the studies until now constituted the only base for the identification of this dye in pieces of art. In this way *Porphyrophora polonica* L. was identified in several antique fabrics, e.g., in the 15th century Italian velvet brocades from the Wawel Cathedral collection [7, 8] and in the 16th century tapestry “Giuseppe fugge dalla moglie di Putifarre” [9]. However, it was only a hypothesis concerning the origin of the dye, unfortunately unproven, as differentiation of species belonging to the same class is a complicated problem [10, 11]. Recently, principal components analysis (PCA) was used to identify different sources of reds, but the results were statistically indistinguishable [12]. Surface-enhanced Raman spectroscopy (SERS) [13–16] or new techniques of ambient mass spectrometry such as direct analysis in real time (DART) [17] also do not seem to be a solution to the problem of differentiation and identification of cochineal species. Despite numerous advantages of these techniques, without separating the components of mixtures they are not able to detect discrete nuances in the composition of minor colorants between different cochineals, especially considering the dominant content of carminic acid in these dyestuffs. However, mass spectrometry coupled with high-performance liquid chromatography has already been used for identification of anthraquinones in American cochineal [7, 18, 19], since various glycosidic compounds may be easily recognized on the basis of the characteristic fragmentation pattern.

The aim of this study was to define compounds of *Porphyrophora polonica* L. by HPLC-DAD-ESI QqQ MS, and consequently to indicate markers for distinguishing of Polish cochineal from other similar dyes of animal origin. The colorants separated on a reverse phase phenyl column were detected spectrophotometrically at 277, 287, 435, 495, and 525 nm, and examined by MS/MS in the negative ion mode. The obtained data formed the basis for the multiple reaction monitoring (MRM) method, used originally for identification of colorants in historical red textiles from the collection of the National Museum in Warsaw.

## Experimental

### Apparatus

Separation and identification of colorants were carried out using the liquid chromatographic system LC 1100 (Agilent Technologies, Wilmington, NC, USA) with spectrophotometric, DAD 1100 (Hewlett-Packard, Waldbronn, Germany), and tandem mass spectrometric detection 6460 Triple Quad LC/MS (Agilent Technologies, Santa Clara, CA, USA). Samples were injected onto a rapid resolution column, Zorbax SB-Phenyl (4.6 × 150 mm, 3.5 *μ*m, 80 Å, Agilent Technologies), using an injection valve, Rheodyne Model 7225i (Cotati, CA, USA) with a 20 *μ*L loop. A precolumn, Zorbax SB-Phenyl (4.6 × 12.5 mm, 5.0 *μ*m, Agilent Technologies), was fitted to protect the main column. The mobile phase, a mixture of (A) 1.5 % (v/v) formic acid in water and (B) methanol, was degassed with the use of Micro Vacuum Degasser 1100 (Agilent Technologies), and the flow-rate was equaled to 0.5 mL min^−1^. Spectrophotometric detection was performed at 277, 287, 435, 495, or 525 nm. Mass spectrometric data were recorded in the full scan and product ion modes of the negative ions for the dye extracts and in the MRM mode for textile fibers. The cycle time of MRM analysis was 500 ms and the width of scanned retention time was 1.2 min. The analyses were controlled by the MassHunter Workstation software (Agilent Technologies). Detailed separation and detection conditions have been described by Lech et al. [19].

The Polish cochineal was dried using a lyophilizer, Alpha 1–2 LD, Martin Christ (Osterode am Harz, Germany). Extraction of colorants was performed with the use of an ultrasonic bath, Branson Model 1210 (Danbury, CT, USA) as well as with a water bath, Memmert WB 10 (Schwabach, Germany), and the extract was separated from the residue using centrifuge MPW-350R, MPW Med. Instruments (Warsaw, Poland).

### Chemicals and materials

Carminic acid of analytical chemical grade was purchased from Fluka (Buchs, Switzerland), kermesic acid was kindly donated by Dr. Ioannis Karapanagiotis (Ormylia Art Diagnosis Center, Greece), and flavokermesic acid was obtained from a mixture of natural product known as lac dye, which was purchased from Kremer-Pigmente (Aichstetten, Germany). Polish cochineal was harvested by Bożena Łagowska and Katarzyna Golan (Department of Entomology, University of Life Sciences, Lublin, Poland) and kindly donated by Jerzy Holc (the head of Conservation Workshop of Historical Textiles at Wawel Royal Castle, Kraków, Poland). American cochineal was purchased from Kremer-Pigmente (Aichstetten, Germany).

Methanol of LC/MS purity was purchased from POCH (Gliwice, Poland), formic acid (>99.5 %) of LC/MS purity, from Fisher Scientific (Fair Lawn, NJ, USA), and hydrochloric acid (35–38 %) of analytical grade, from AppliChem (Darmstadt, Germany). Demineralized water was made using Milli-Q system Model Millipore Elix 3 (Molsheim, France). Examined fibers were taken from seven Polish textiles dated to the 17th–19th century and provided by Ewa Orlińska-Mianowska from the Textile Division of the National Museum in Warsaw.

### Standards solutions

A sample of 2 mg of each standard preparation was dissolved in 10 mL of methanol. The obtained solutions were filtered over a 0.45 *μ*m PET syringe filter (PPHU Q3 S.C., Brzeziny, Poland). The first five drops were discarded, and only the remaining parts of the filtrates after dilution were used for the analysis.

### Extraction of raw preparations

Two mg of lyophilized and ground Polish cochineal was extracted with 250 *μ*L of methanol. The solution was kept in an ultrasonic bath for 15 min, and in a water bath (at 60 °C) for the next 15 min, then filtered over a 0.22 *μ*m PET syringe filter. Dilution of the extract with water (1:1, v/v) resulted in immediate precipitation of a white solid, which was separated by centrifugation (10,000 rpm, 70 min, 21 °C) through Amicon Ultra-0.5 Centrifugal Filter Unit with Ultracel-3 membrane shut-off compounds above 3 kDa (Merck Millipore Ltd., Carrigtwohill, Ireland). The obtained solution was analyzed as described above.

Extract of American cochineal for comparative analysis was prepared by dissolving 4 mg of dried powder in 1 mL of methanol. In this case, centrifugation was not required as precipitation did nor form. The obtained solution, before further analysis steps, was diluted 30 times with a mixture of methanol and water (1:1, v/v).

### Extraction of fibers

Fibers, 0.2–0.3 mg, were treated with two extraction procedures. In the first one, samples were extracted with 50 *μ*L of a mixture of methanol, water, and concentrated formic acid (9:8:3, v/v/v; such composition was optimized for the most efficient extraction of *O*-glycosides). They were kept in an ultrasonic bath for 20 min and then in a water bath (at 60 °C) for the next 25 min. The obtained extracts were separated from the fiber and diluted with 25 *μ*L of a mixture of methanol and water (2:3, v/v). The second extraction was performed with 50 *μ*L of a mixture of methanol and 37 % hydrochloric acid (17:3, v/v). The obtained extracts were separated from the fiber and diluted with 50 *μ*L of water.

The extraction procedure was optimized for fibers dyed with weld, Persian berries, madder, and American cochineal. The optimization included the following steps: (a) the type of extractant used (methanol, methanol–water, methanol-acetonitrile, dimethyl sulfoxide), (b) the amount of formic or hydrochloric acid (0.5/20, 1/20, 2/20, or 3/20 of volume), (c) time of exposure to the ultrasound (5, 10, 20, or 30 min), and (d) the temperature and time of extraction (50, 60, 70 or 80 °C for 10, 25, 40, or 55 min). The most effective extraction of *O*-glycosides from the dyed fibers has been obtained for a mixture of methanol, water, and formic acid, and the procedure described above.

## Results and discussion

### Polish cochineal

The aim of the study was to create a fingerprint of color compounds present in Polish cochineal and to find its markers, which would allow distinguishing of this dye from other reds of animal origin, primarily American cochineal. For this reason, HPLC-UV–VIS-ESI QqQ MS system was used. The compounds separated on a reverse phase phenyl column were detected spectrophotometrically at various wavelengths as well as by the use of mass spectrometer in the negative ion mode under various acquisition conditions. MS detection performed in the full *m/z*-scans allowed selecting of deprotonated quasi-molecular ions ([M − H]^−^) for further MS/MS analysis in the product ion mode with the use of different CID energies. The *C*-glycosides decomposed through cross-ring cleavages giving ^*k,l*^X_*j*_ ions (superscripts *k* and *l* indicate cleavage links in carbohydrate rings and subscript *j* refers to the number of interglycosidic bonds, in accordance with the system proposed by Domon and Costello [20]); meanwhile, *O*-glycosides are broken down to Y_*j*_ ions by dissociation of weak glycoside bonds [21]. Thus, at the same time these fragments provide information about the masses of aglycones and their structures as well as indirectly about the presence of functional groups [19].

#### Colorants already reported

**Fig. 1:**
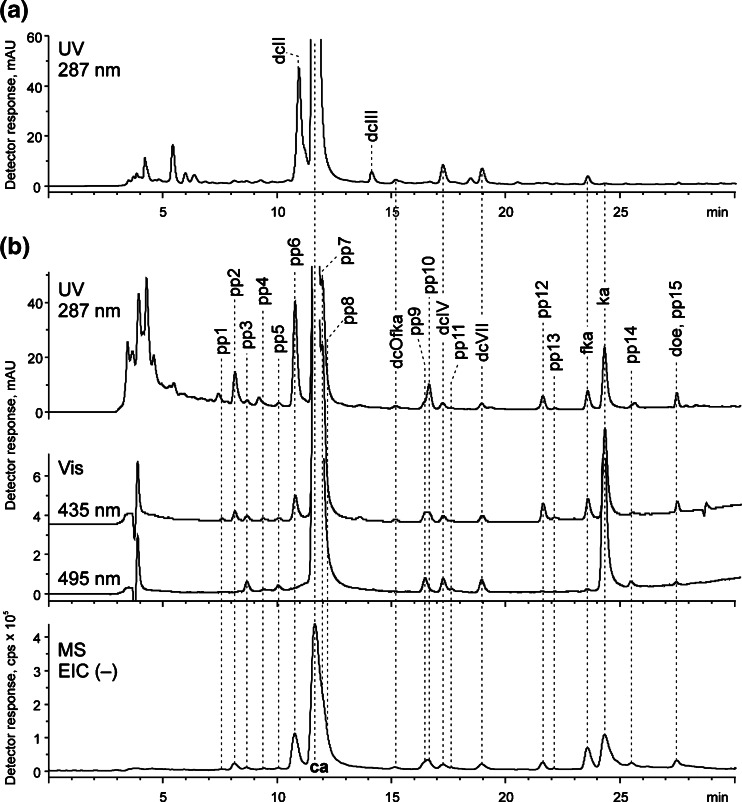
**a** UV chromatogram (287 nm) of extract from American cochineal, **b** UV–VIS (287, 435, and 495 nm) and MS chromatograms (extracted negative ion) of extract from Polish cochineal (cf. Table 1)

Chromatograms of the extracts of Polish and American cochineal (obtained with MS and spectrophotometric detection) are apparently almost identical (Fig. 1; retention times of all separated compounds, the *m/z* values of their ions as well as their structural characterization are presented in Table 1). They show the most intensive peak of carminic acid (*C*-glucopyranoside of kermesic acid) and smaller signals of its two isomers, dcIV and dcVII, as well as peaks of kermesic (ka) and flavokermesic acid (fka). Their MS/MS spectra corresponded with the data presented previously [7, 19, 22] [see Electronic Supplementary Material (ESM) Fig. S1]. Carminic acid loses carbon dioxide (44 Da) from the carboxyl group and *C*-glucoside moiety decomposes via cross-ring cleavages (losses of 90, 120, and 148 Da) resulting in the formation of ions at *m/z* 357 [^0,3^X – H − CO_2_]^−^, 327 [^0,2^X – H − CO_2_]^−^, and 299 [^0,1^X – H − CO_2_]^−^. The aglycones dissociated by the subsequent losses of CO_2_ (44 Da) and CO (28 Da). Moreover, the chromatograms also show one more peak corresponding to 6-*O*-*α*-D-glucopyranoside of flavokermesic acid (dcOfka). Its occurrence has already been reported in the case of American cochineal [19, 20], but so far it has not been found in Polish cochineal.

**Table 1:** Anthraquinone compounds extracted from *Porphyrophora polonica* L.

	t_*R*_, min	[M − H]^−^, *m/z*	Characteristic					
			Glycoside		Aglycone			Additional information
pp1	7.7	653	*O*-, *O*-		ka			
pp2	8.1	593	*O*-, *O*-				doe	
pp3	8.6	653		di-*C*-	ka			
pp4	9.4	653	di-*O*-		ka			
pp5	10.0	653	*O*-	*C*-	ka			
pp6 (ppI)	10.7	475	*O*-			fka		
ca	11.6	491		*C*-	ka			carminic acid (7-*C*-*α*-D-glucopyranoside of kermesic acid)
pp7 (ppII)	12.0	491	*O*-		ka			
pp8	12.2	475	*O*-			fka		
dcOfka	15.2	475	*O*-			fka		6-*O*-*α*-D-glucopyranoside of flavokermesic acid [17]
pp9	16.4	491	*O*-		ka			
pp10	16.6	431	*O*-				doe	
dcIV	17.2	491		*C*-	ka			7-*C*-*α*-D-glucofuranoside of kermesic acid [17]
pp11	17.6	533		*C*- ^a^	ka			
dcVII	18.9	491		*C*-	ka			7-*C*-*β*-D-glucofuranoside of kermesic acid [17]
pp12	21.5	431	*O*-				doe	
pp13	22.1	431	*O*-				doe	
fka	23.5	313	–––	–––				flavokermesic acid
ka	24.3	329	–––	–––	ka			kermesic acid
pp14	25.4	589		*C*- ^a^	ka			
pp15	27.4	617		*C*- ^a^	ka			
doe	27.4	269	–––	–––				deoxyerythrolaccin (decarboxylated flavokermesic acid)

^a^ *C*-hexoside with additional substituent

The six peaks discussed above appear in the chromatograms of both cochineals. However, the chromatogram of the extract of *Porphyrophora polonica* L. registered by spectrophotometric detector at 435, 495, and 525 nm is much more rich, and several other signals can be clearly distinguished (compounds pp1-pp15 and doe). According to the best knowledge of the authors, such set of data has not been reported so far.

#### Colorants unknown so far

##### C-glycosides

**Fig. 2:**
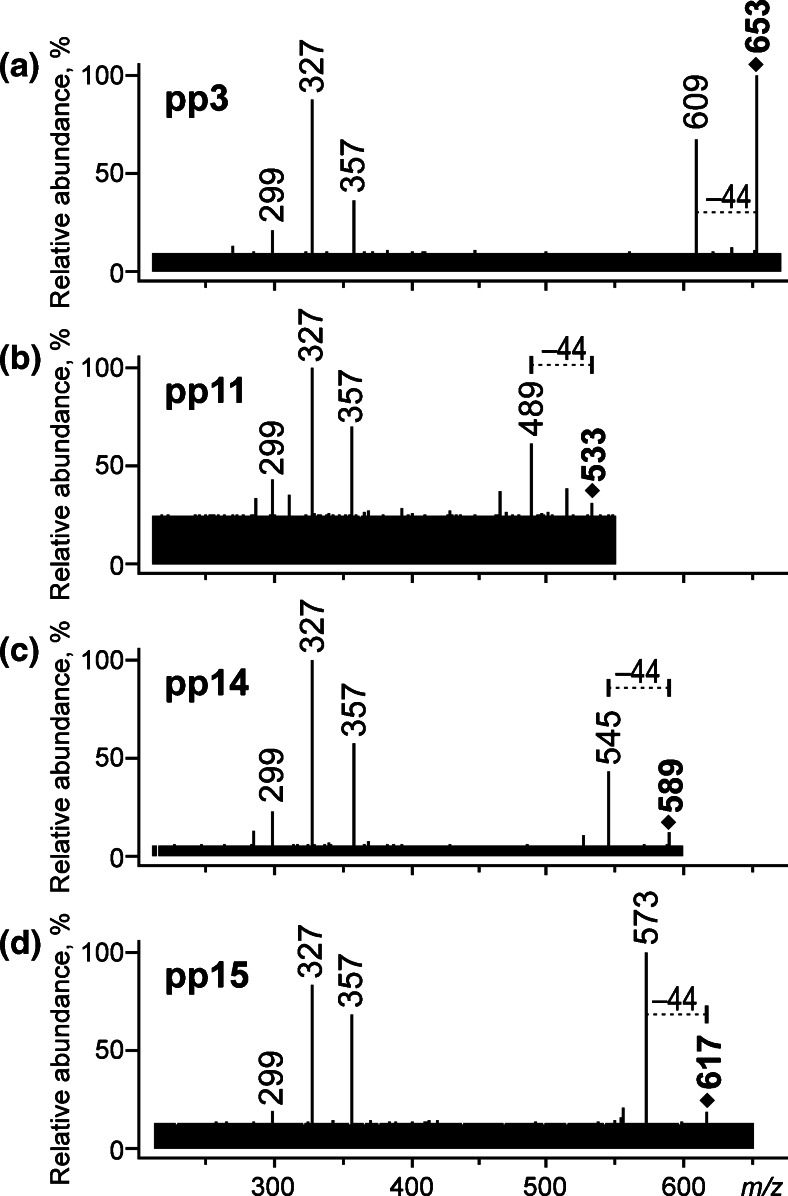
ESI QqQ MS product ion spectra (mother ions – [M − H]^−^, CE 25 V) of *C*-glycosides

Careful analysis of the MS/MS spectra allowed finding among the discovered colorants of four *C*-glycosides that are carboxylic acids at the same time (pp3, pp11, pp14, and pp15). They were identified thanks to the signals identical to those of carminic acid corresponding to cross-ring fragmentation of *C*-hexoside moiety (at *m/z* 357, 327, and 299) as well as the loss of CO_2_ (44 Da) from carboxylic groups (Fig. 2). However, the *m/z* values of their quasi-molecular ions are higher than the one of carminic acid (*m/z* 491), and the differences are 162 Da (pp3, [M − H]^−^ at *m/z* 653), 42 Da (pp11, *m/z* 533), 98 Da (pp14, *m/z* 589), and 126 Da (pp15, *m/z* 617). It proves that each compound contains additional substituent bonded via a hydroxyl group in position between C-4' and C-6' of its sugar moiety (presence of additional groups in these positions does not affect further fragmentation). Nominal values of these differences allow indication of the following formulas for the substituents: a hexose moiety, probably bonded to C-6’ (pp3), C_3_H_7_ (pp11), C_4_H_3_O_3_, or C_5_H_7_O_2_ (pp14), and C_6_H_7_O_3_ (pp15).

##### O-glycosides

**Fig. 3:**
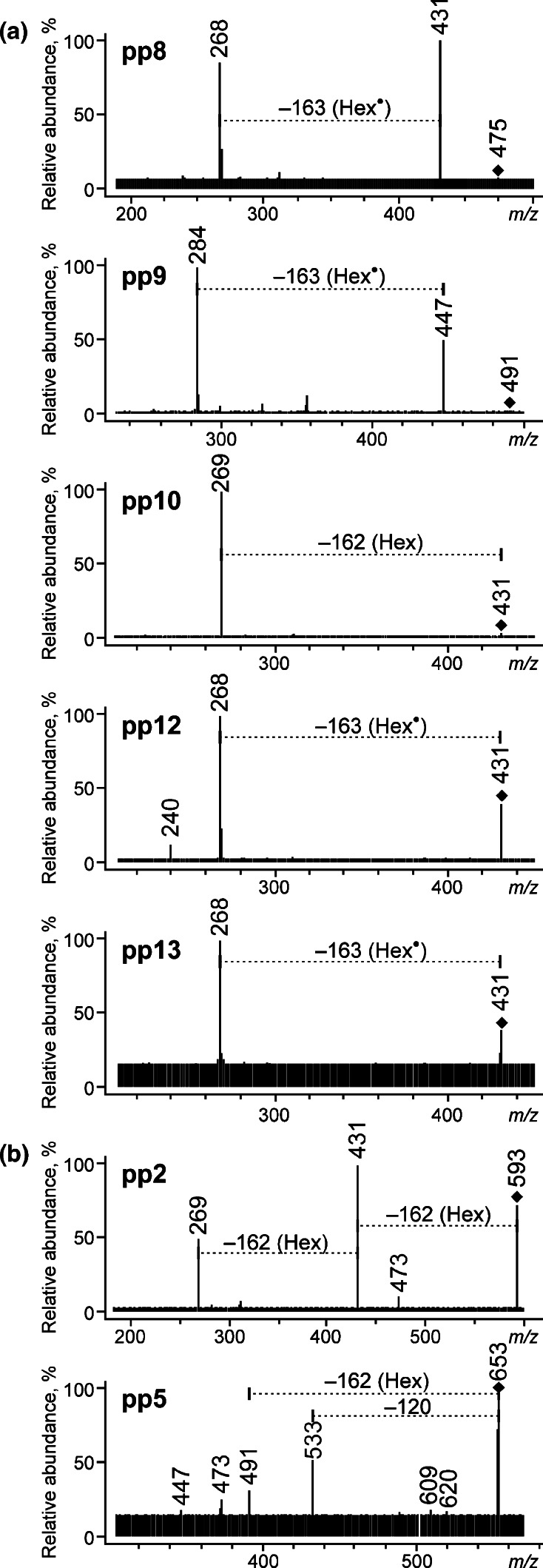
ESI QqQ MS product ion spectra (mother ions – [M − H]^−^, CE 25 V) of (**a**) *O*-glycosides and (**b**) diglycosides

Characteristic losses of 162 Da observed in some of the MS/MS spectra (Figs. 3a, 5a, and 6b) corresponded to dissociation of a hexose moiety, Hex (in this case probably a glucose, Glc). These results allowed identification of eight of the separated compounds as *O*-hexosides (i.e., pp6, pp7, pp8, pp9, pp10, pp12, and pp13 reported herein for the first time) as well as already mentioned dcOfka. The first seven compounds can be classified into three groups according to the *m/z* values of their quasi-molecular ions: *O*-hexosides of kermesic acid (pp7 and pp9) with the [M − H]^−^ ions at *m/z* 491, *O*-hexosides of flavokermesic acid (pp6 and pp8) – [M − H]^−^ at *m/z* 475 and *O*-hexosides of deoxyerythrolaccin (pp10, pp12 and pp13) – [M − H]^−^ at *m/z* 431. The compounds belonging to the first and to the second group gave two characteristic signals: the first one corresponding to the loss of carbon dioxide ([M – H − CO_2_]^−^ at *m/z* 447 and 431, respectively) and the second one to further dissociation of a hexose moiety ([M – H − CO_2_ − Hex]^−^ or [M – H − CO_2_ − Hex]^−•^ at *m/z* 285 or 284, and 269 or 268, respectively). The colorants from the third group gave only one ion – [M – H − Hex]^−^ or [M – H − CO_2_ − Hex]^−•^ (*m/z* 269 or 268).

Careful analysis of UV–VIS spectra of selected *O*-glycosides allows formulation of hypothesis concerning position of glycosidic substituents in their anthraquinone moiety. Spectrophotometric spectra of anthraquinones extracted from animal reds show three characteristic bands between 230 and 550 nm related to benzoyl and quinoid absorption [23–25]. As it was previously reported [25, 26], electron-releasing groups (e.g., −OH, −OCH_3_ or –NH_2_) situated in the α-positions (1-, 4-, 5-, 8-) of anthraquinone rings are responsible for absorption at longer wavelengths as well as for an increase in its intensity. These substituents enable the existence of resonance forms and the creation of the intramolecular hydrogen bond, which stabilize the excited states. Thus, the involvement of the hydroxyl groups present in these positions (an auxochrome) and the formation of a *O*-glycosidic bond cause changes in the spectra of these glycosides.

**Fig. 4:**
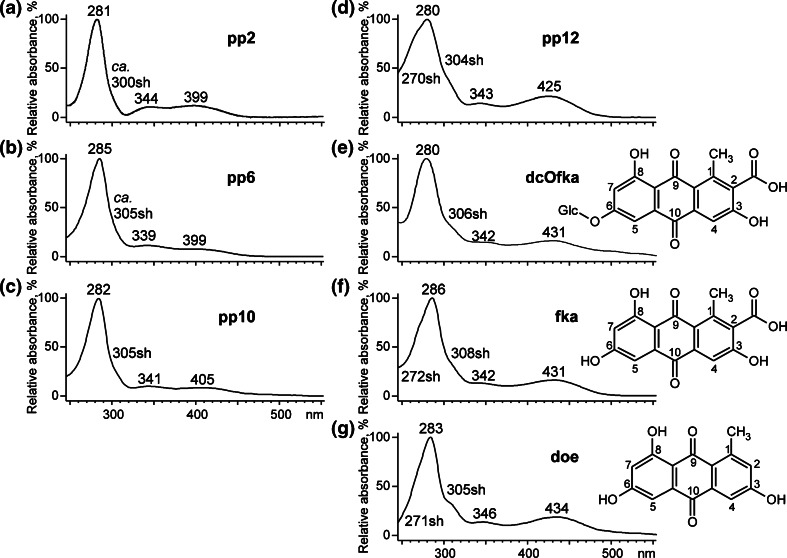
Absorption spectra of (**a**) pp2, (**b**) pp6, (**c**) pp10, (**d**) pp12, (**e**) dcOfka, (**f**) flavokermesic acid (fka), and (**g**) deoxyerythrolaccin (doe)

Slight absorption maxima at 399–405 nm of pp2, pp6, and pp10 (Fig. 4) prove that the hexose moiety is bonded with an aglycone via an α-hydroxyl group (in these cases probably at the 8-C position). A much more intense band of pp12 at 425–434 nm, which is similar to deoxyerythrolaccin or flavokermesic acid, suggests that this colorant forms glycosidic bonds probably via a hydroxyl group in the position 3- or 6-C, as it is in the case of dcOfka.

##### Diglycosides

The obtained data confirm presence of five diglycosides among the identified compounds. Four of them (pp1, pp3, pp4, and pp5) form the quasi-molecular ions at the same *m/z* value – 653. The structure of pp3 has already been discussed above in the section “C*-glycosides”*, and the colorant has been identified as *C*-dihexoside of kermesic acid. The MS/MS spectra of pp1 and pp4 show ions at *m/z* 609 corresponding to the loss of CO_2_. The first compound also gives two intense signals at *m/z* 447 and 285 corresponding to loss of one and two hexose moieties ([M – H − CO_2_ − Hex]^−^ and [M – H − CO_2_ − 2Hex]^−^). These results suggest that each of the sugar moieties is bound to different atoms of kermesic acid via *O*-glycosidic bonds (di-*O*,*O*-hexoside of kermesic acid).

Apart from the ion at *m/z* 609 ([M – H − CO_2_]^−^), pp4 forms only the ion registered at *m/z* 285 corresponding to simultaneous loss of two hexose moieties together. Such fragmentation allows its identification as *O*-dihexoside of kermesic acid.

The MS/MS spectrum of pp5 (Fig. 3b), the last compound of quasi-molecular ion at *m/z* 653, is ambiguous; on the one hand, it show signals at *m/z* 491 (loss of 162 Da) and *m/z* 533 (loss of 120 Da), and on the other, it does not contain a clear signal corresponding to loss of CO_2_ (44 Da). It forms a basis for a hypothesis that pp5 is dihexoside containing *C*- as well as *O*-bonded sugars, wherein a carboxyl group is absent or linked to another substituent, as it can be suggested by the lack of decarboxylation. However, a strict structure of this compound cannot be proposed in light of the obtained results.

Compound pp2 gives the [M − H]^−^ ion at *m/z* 593, MS/MS spectrum of which (Fig. 3b) shows a fragmentation characteristic for di-*O*,*O*-hexosides, which is consecutive losses of two hexose moieties ([M – H − Hex]^−^ at *m/z* 431 and [M – H − 2Hex]^−^ at *m/z* 269 are observed). Moreover, the lack of CO_2_ loss and the [Y_0_ − H]^−^ ion at *m/z* 269 indicate that its aglycone is deoxyerythrolaccin.

##### Non-glycosidic colorant

Apart from the aforementioned novel glycoside compounds, another constituent (co-eluted with pp15 at 27.4 min) was found in the extract from Polish cochineal. Its [M − H]^−^ ion at *m/z* 269 gives the MS/MS spectrum almost identical to that of flavokermesic acid, but does not consist of a signal corresponding to the loss of CO_2_. This similarity allowed its identification as deoxyerythrolaccin (doe, flavokermesic acid without the carboxylic group). This hypothesis seems to be justified by the fact that its *O*-glycosides (pp2, pp10, pp12, and pp13) are also identified in the extract.

#### Hitherto unknown markers of Polish cochineal

**Fig. 5:**
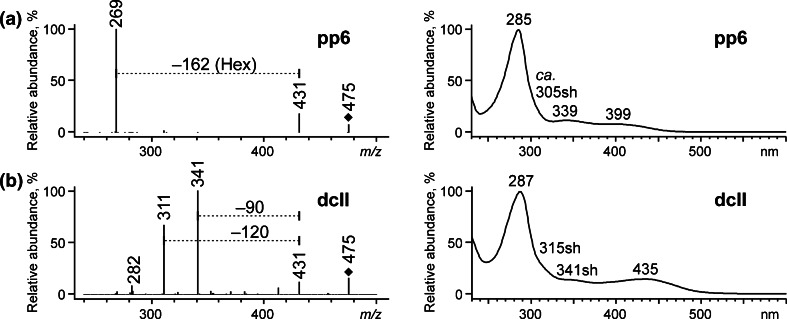
ESI QqQ MS product ion spectra (mother ions – [M − H]^−^, CE 20 V) and absorption curves of (**a**) pp6, (**b**) dcII; pp6 was found in the extract obtained from Polish cochineal, dcII – from American cochineal

Apparently identical chromatograms of extracts from American and Polish cochineals in the range of 10.7–12.0 min are in fact crucial for differentiation between these two natural red dyestuffs. The chromatogram of the extract obtained from Polish cochineal consists of peak at t_*R*_ 10.7 min, whereas from the American one, at 10.8 min. Despite this small difference, these peaks correspond to different compounds of the same molecular mass but of different spectral characteristics (Fig. 5). Careful analysis of the MS/MS data supported by UV–VIS measurements allowed identifying them as pp6, *O*-hexoside of flavokermesic acid (t_*R*_ 10.7 min), and dcII, 7-*C*-α-D-glucopyranoside of flavokermesic acid (t_*R*_ 10.8 min). Because of a relatively intensive chromatographic peak of pp6, this compound was proposed as the marker that would allow distinguishing between Polish cochineal and other cochineal species. It also has to be noted that comparison of its UV–VIS spectrum with the one of ppI presented by Wouters and Verhecken [5] leads to the conclusion that pp6 and ppI are in fact the same compound, postulated as a precursor of flavokermesic acid.

**Fig. 6:**
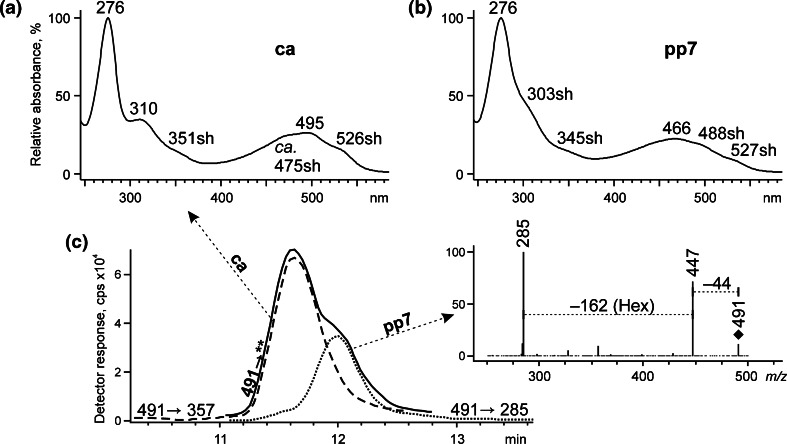
ESI QqQ MS product ion spectra (mother ions – [M − H]^−^ at *m/z* 491, CE 20 V) and/or absorption curves of (**a**) carminic acid and (**b**) pp7; (**c**) ESI MS chromatograms reconstructed for mother ion at *m/z* 491, and MRM pairs at *m/z* 491 → 327 and *m/z* 491 → 285

Apart from pp6, the extract of Polish cochineal contains another colorant of relatively large concentration, pp7. This compound has been reported previously by Wouters and Verhecken [5, 6] as ppII – a precursor of kermesic acid, but its structure has not been proposed so far, since its co-elution (t_*R*_ 12.0 min) with carminic acid (t_*R*_ 11.6 min) made registration of its spectrophotometric spectrum impossible. In the present study, MS/MS complimented with UV–VIS data (Fig. 6) allowed identification of pp7 as *O*-glycoside of kermesic acid.

### Textiles analysis

Analysis of fibers taken from historical fabric belonging to the National Museum in Warsaw was performed by the MRM method developed for this purpose. The MRM pairs have been chosen on the basis of the obtained MS/MS spectra. Tandem mass spectrometry in the MRM mode is a very sensitive technique; thus, main colorants of a dye can be detected even if a thread is of trace amount (less than 0.1 mg). In the case of trace compounds, such as pp6, the required amount of sample depends on intensity of its color and/or on a degree of its degradation.

Carminic acid is the main colorant present in all examined extracts obtained using the mixture of methanol, water, and formic acid. They also contain many other compounds (e.g., ellagic acid, dcIV, dcVII, as well as kermesic and flavokermesic acid). Peaks of the last two compounds are much higher in the chromatograms obtained for extracts of threads taken from SZT.1884, SZT.2007, SZT.2550, and SZT.2716. Moreover, these chromatograms also show a bimodal peak observed just before the peak of carminic acid and registered for the mother ion at *m/z* 475.

The reconstruction of its MRM chromatograms indicates that it corresponds to pp6 (ppI) (*m/z* 475 → 269) and dcII (*m/z* 475 → 341, 311, 282) as well, the isomers eluting at approximately the same time. Since pp6 (ppI) has already been proposed by us as a marker of Polish cochineal, its absence in other extracts suggests that those fibers were probably dyed with American cochineal species (Table 2). However, because of almost the same composition of Polish and Armenian cochineals [5, 6], the obtained results do not allow definitive distinction of these two *Porphyrophora* species.

**Table 2:** Colorants identified by HPLC-DAD-ESI MS/MS (MRM method) in extracts from textiles from the collection of the Warsaw National Museum

Inventory number of textile	Dating	Origin	Colorants	Identified dye
SZT.1320	the 19th c.	a carpet in floral – red wool thread from knot; Polish origin	dcII, ca, ea, dcIV, dcVII, fka, ka	American cochineal
SZT.1463/1-2	half of the 70's, the 16th c.	a fragment of the trail from a linen altar table-cloth of Queen Anna Jagiellon's foundation – red silk thread from embroidery; Polish origin (probably courtly workshop)	dcII, ca, dc3, ea, dcIV, dcVII, fka, ka	American cochineal
SZT.1740	the 16th c.	a linen church table-cloth embroidered with combined vortical rosettes, flat embroidery – red silk thread from embroidery; Polish origin	ca, dc3, dc5, q, ea, dcIV, dcVII, fka, ka, a	American cochineal
SZT.1884/a-b	the end of the 17th c.	a fragment of the chasuble embroidered in floral (flowers and twigs) – red silk thread from embroidery; Polish origin	ga, pp6, dcII, ca, dcOfka, ea, dcIV, dcVII, fka, ka, e	Polish cochineal
SZT.2007	the 18th c.	a fragment of a column in a chasuble embroidered in flowers – red silk thread from embroidery; Polish origin	ga, pp6, dcII, ca, dc3, dcOfka, pp10, ea, dcIV, dc7, dcVII, fka, ka	Polish cochineal
SZT.2550	1695	a chasuble embroidered with motifs of the floral flagellum on the sides and platters of fruit or flowers in column, made in the workshop of Poznan, embroiderer Franciszek Antoni Wojciechowski – red silk thread from a column; Polish origin (Poznan)	ga, pp6, dcII, ca, dcOfka, ea, dcIV, dcVII, fka, ka	Polish cochineal
SZT.2716	the 18th c.	a men's vest in a cherry-colored, embroidered in a pattern of stylized flowers – red silk thread from satin; Polish origin (Silesia)	ga, is, pp6, dcII, ca, dc3, dcOfka, pp10, ea, dcIV, dc7, dcVII, l, fka, ka	Polish cochineal

a: apigenin; ca: carminic acid; e: emodin; ea: ellagic acid; fka: flavokermesic acid; is: isatin; ka: kermesic acid; l: luteolin; q: quercitrin

It must be noted that the proposed way for the identification of Polish cochineal is effective only if the extraction of colorants from the thread is carried out under mild conditions [27] (with the use of methanol–water–formic acid mixture). Hence, when hydrochloric acid is used instead of formic acid, pp6 is not observed in the chromatogram. This is due to decomposition of pp6 (ppI) by hydrochloric acid that hydrolyzes *O*-glycosidic bonds.

## Conclusions

The result of the present study clearly indicates that tandem mass spectrometric detection coupled with high-performance liquid chromatography is essential for the unequivocal identification of Polish cochineal. The MS/MS fragmentation experiments enable fast and reliable evaluation of even limited number of samples.

Chemical differentiation between red animal dyes obtained from cochineal scale insects such as Polish and American cochineal has been extremely difficult so far, since they have similar composition of their anthraquinone compounds. Examination of the extract obtained from Polish cochineal by HPLC-DAD-ESI QqQ MS allowed identification of 22 color compounds; the structures of 16 among them have not yet been proposed. Most of them were *O*- or *C*-hexosides of kermesic and flavokermesic acids, or deoxyerythrolaccin. Five of them (i.e., pp2, pp6 (ppI), pp7 (ppII), pp10, and pp12) are proposed as specific markers of Polish cochineal because of their complete absence in American cochineal and the relatively high intensity of their chromatographic peaks. Hence, it can be assumed that these compounds can also participate in the dyeing process by bonding with the fiber via various mordants. As a consequence, they can be detected and identified using advanced techniques, such as HPLC-UV–VIS or HPLC-ESI MS/MS. However, they can be observed in the extracts only when mild isolation procedure (with addition of formic acid instead of previously used hydrochloric one) is carried out. Particular attention has to be paid to pp6 (ppI, *O*-hexoside of flavokermesic acid), an isomer of dcII (*C*-glucoside of flavokermesic acid, present mainly in American cochineal). Despite nearly the same retention times, they were differentiated on the basis of their different mass and spectrophotometric spectra. Even though dcII was not noted in Polish cochineal, this compound always accompanied pp6 in the extracts obtained from the historical threads. Nonetheless, dcII is not a marker of any dye, and may be present in *Dactylopius* as well as *Porphyrophora* species [2, 3, 12], and hence its presence does not exclude the use of Polish cochineal, whereas pp6 precludes the American one.

It was assumed that a substantially higher content of kermesic and flavokermesic acids (reported in earlier studies) in Polish cochineal compared with that of American cochineal can result from the fact that in the first preparation mainly *O*-glycosides are present, whereas in the second one, *C*-glycosides are predominant [19]. The *O*-glycosidic bond is much weaker than the *C*-glycosidic one; thus strong mineral acid causes its hydrolysis and formation of free aglycones more easily. Consequently, kermesic and flavokermesic acids were determined in significantly larger amount in Polish than in American cochineal.

However, according to Wouters and Verhecken [5, 6], the composition of colorants in Polish and Armenian cochineals is very similar; in both dyes pp2, pp6, pp7, pp10, or pp12 may be found.

It should be noted that the partial-least squares discriminant analysis (PLS-DA) applied to model UHPLC-PDA data for discrimination of cochineal insect dyes was published recently [28]. However, this statistical method requires a large number of reference samples to create a correct model, which seems to be particularly difficult given the limited availability of Polish cochineal. The present study demonstrates that detection of minor but characteristic compounds can also be an efficient tool for identification of Polish cochineal in the historical samples, especially if access to the respective number of dye samples is restricted. Nevertheless, if only appropriate reference materials are available, further studies using PLS-DA would be undertaken in order to validate the coherence of these two independent approaches.

## Electronic supplementary material

Below is the link to the electronic supplementary material.

ESM 1 (PDF 148 kb)
