# Supplementary material for: Identification of Polish cochineal (Porphyrophora polonica L.) in historical textiles by high-performance liquid chromatography coupled with spectrophotometric and tandem mass spectrometric detection
Source: Anal Bioanal Chem. 2016 Mar 2;408:3349–58. doi: 10.1007/s00216-016-9408-0 (PMC4830872; doi:10.1007/s00216-016-9408-0)
Supplement: Supplementary file 1 — (PDF 148 kb) [file 216_2016_9408_MOESM1_ESM.pdf]

Analytical and Bioanalytical Chemistry

Electronic Supplementary Material

Identification of Polish cochineal (*Porphyrophora polonica* L.) in historical textiles by high-performance liquid chromatography coupled with spectrophotometric and tandem mass spectrometric detection

Katarzyna Lech, Maciej Jarosz

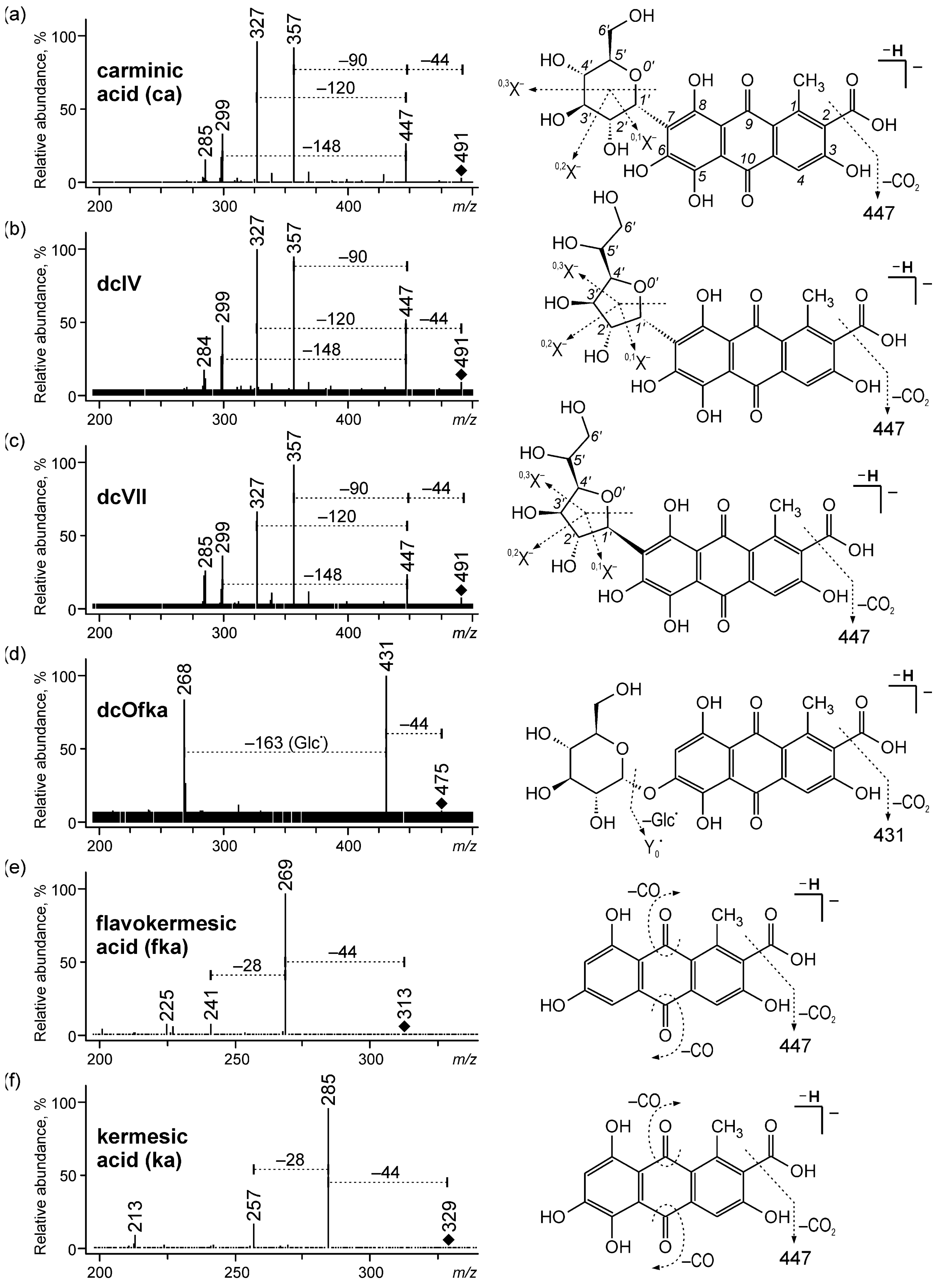

Fig. S1 ESI QqQ MS product ion spectra (mother ions – [M–H]$^-$, CE 25 V) and fragmentation pathways of (a) carminic acid, (b) dcIV, (c) dcVII, (d) dcOfka, (e) flavokermesic acid, and (f) kermesic acid
